# CyberGenomics: Application of Behavioral Genetics in Cybersecurity

**DOI:** 10.3390/bs11110152

**Published:** 2021-11-01

**Authors:** Ingrida Domarkienė, Laima Ambrozaitytė, Linas Bukauskas, Tautvydas Rančelis, Stefan Sütterlin, Benjamin James Knox, Kaie Maennel, Olaf Maennel, Karen Parish, Ricardo Gregorio Lugo, Agnė Brilingaitė

**Affiliations:** 1 Department of Human and Medical Genetics, Institute of Biomedical Sciences, Faculty of Medicine, Vilnius University, LT-08661 Vilnius, Lithuania; laima.ambrozaityte@mf.vu.lt (L.A.); tautvydas.rancelis@mf.vu.lt (T.R.); 2 Cybersecurity Laboratory, Institute of Computer Science, Vilnius University, LT-08303 Vilnius, Lithuania; linas.bukauskas@mif.vu.lt (L.B.); agne.brilingaite@mif.vu.lt (A.B.); 3 Faculty of Health, Welfare and Organisation, Østfold University College, NO-1757 Halden, Norway; stefan.sutterlin@hiof.no (S.S.); benjamin.j.knox@ntnu.no (B.J.K.); ricardo.g.lugo@ntnu.no (R.G.L.); 4 Centre for Digital Forensics and Cyber Security, Tallinn University of Technology, EE-19086 Tallinn, Estonia; kaie.maennel@taltech.ee (K.M.); olaf.maennel@taltech.ee (O.M.); 5 Department of Information Security and Communication Technology, Norwegian University of Science and Technology (NTNU), NO-2802 Gjøvik, Norway; karen.parish@ntnu.no; 6 Center for Cyber and Information Security, Norwegian University of Science and Technology (NTNU), NO-2802 Gjøvik, Norway

**Keywords:** cybersecurity, risk assessment, genetic architecture, behavior genetics, complex traits, genome-wide association study (GWAS), human factor, human behavior, stress genomics

## Abstract

Cybersecurity (CS) is a contemporary field for research and applied study of a range of aspects from across multiple disciplines. A cybersecurity expert has an in-depth knowledge of technology but is often also recognized for the ability to view technology in a non-standard way. This paper explores how CS specialists are both a combination of professional computing-based skills and genetically encoded traits. Almost every human behavioral trait is a result of many genome variants in action altogether with environmental factors. The review focuses on contextualizing the behavior genetics aspects in the application of cybersecurity. It reconsiders methods that help to identify aspects of human behavior from the genetic information. And stress is an illustrative factor to start the discussion within the community on what methodology should be used in an ethical way to approach those questions. CS positions are considered stressful due to the complexity of the domain and the social impact it can have in cases of failure. An individual risk profile could be created combining known genome variants linked to a trait of particular behavior using a special biostatistical approach such as a polygenic score. These revised advancements bring challenging possibilities in the applications of human behavior genetics and CS.

## 1. Introduction

Cybersecurity (CS) is a contemporary field for research and applied study of a range of aspects from across multiple disciplines. The guidelines for CS curricula distinguish a knowledge area of Human Security (HS) and Organizational Security (OS). OS relates to laws, regulations, standards to support risk management, planning, governance, and risk assessment concerning insider threats that come from authorized access to sensitive data and systems. These tasks are accompanied by the challenges of coping with stress, fatigue, and the need for effective teamwork. HS covers topics such as CS awareness, social engineering attacks, and abilities related to human misbehavior, e.g., the ability to implement measures to detect and mitigate social engineering attacks and discuss the importance of risk perception in the context of mental models of CS and privacy.

CS positions are considered stressful due to the complexity of the domain and the social impact it can have in cases of failure. The defense must be timely, as errors might cause severe effects. Attack models and vectors are becoming more advanced due to the development of technologies. Many soft skills lead to complex human behavior. One of the soft social skills shaping factors might be genetic factors. Almost every human behavioral trait is a result of many genome variants in action altogether with environmental factors. The Human Genome Project [1] prompted the advancement of the genome, transcriptome, and epigenome sequencing technology and analysis methodology. The development of the technologies not only reduced the cost of genomic data generation but also introduced many approaches to studying the interconnectedness of phenome-wide and genome-wide coherence. A big leap was genome-wide association studies (GWAS) that identified hundreds of genome variants related to particular behavioral traits. Recent technological developments in approaches, i.e., machine learning algorithm approaches, have aided in the analysis of phenome-wide association studies (PheWAS), expression quantitative trait loci (eQTL) analysis, or whole-exome, whole-genome analysis. These advancements bring challenging possibilities in the applications of human genetics and CS.

The possible underlying genetic background of physical and psychological traits, fundamental for human performance under stressful conditions, might also be the same mechanisms responsible for substance abuse, cardiovascular diseases, bipolar disorder, as well as soft skills such as the ability to communicate, cope with fatigue, pressure, stress, remain attentive, assertive, consistent, maintain sharp cognition and incident response. An individual risk profile could be created combining known genome variants linked to a trait of particular behavior using a special biostatistical approach such as a polygenic score. With this knowledge, a CS specialist could become more aware of personal characteristics and environmental conditions and learn to mitigate potential threats. Therefore, genetic information can become an excellent tool for self-knowledge, which can ensure better CS performance.

This paper explores how CS specialists are both a combination of professional computing-based skills and genetically encoded traits. We review methods that help to identify aspects of human behavior from genetic information. Behavior genetics addresses the interdisciplinary effort to establish causal links between genomic loci and human behavioral traits and neural mechanisms. A CS specialist who understands the risks of his behavior can better adapt to adverse environmental conditions and cope with risk factors through well-rehearsed techniques.

According to recent evolutionary-inspired theories (i.e., differential susceptibility, biological sensitivity to context), humans differ substantially in their sensitivity to contextual factors, with some more susceptible to environmental influences than others. Importantly, these theories suggest that heightened sensitivity predicts both the reactivity to adverse contexts as well as the propensity to benefit from supportive features of positive environments [2]. The integration of genomic approaches into the analysis of social traits might deepen the understanding of biology for human behaviors. Furthermore, risks related to the behavior of the person may be determined by genomics and considered in the CS field that is typically associated with technological sciences.

The primary idea of this research was to aid the CS field as it became essential in all parts of life and increasingly problematic year on year. Our main goal was to spark the scientific discussion of whether the genetic component could advance the CS topic in any way. We believe this discussion could inspire further research studies exploring interdisciplinary approaches in CS.

The article addresses the CS field as a complex discipline with multiple layers. We deconstruct the CS specialist as a material (naturally/ genetically determined) and non-material (psychologically determined) entity. Then, we map this entity to CS competences required to conduct everyday tasks with stress as a psychological factor. All the structural prerequisites for the development and functioning of the psyche are genetically coded and controlled. This could be extrapolated universally in other research areas related to human behavior as well.

In this paper, we discuss the complex matter which was, and still is, the main limiting factor of behavior genetics applications—the complexity of behavioral traits, methodologies, and technologies dedicated to the research of genetic architecture of a trait, ethical aspects, and competence frameworks.

## 2. Genomic Factors

The genetic architecture of a phenotype refers to an entire complement of underlying genetic factors, including their number, variant frequencies, and effect sizes of contributing variants. The variation spectrum underlying complex phenotypes includes at least three major classes of DNA variants: common single-nucleotide variants (common SNVs or ‘SNPs’, allele frequencies 1%), rare single-nucleotide variants (rare SNVs, allele frequencies <1%), structural variants, including copy number variants (CNVs), insertions/deletions, and balanced translocations. In addition to inherited variation, rare variants can occur de novo, arising in a parental gamete, a fertilized embryo, or the developing fetus [3].

Until recently, the success of the prediction of genetic effects was limited due to the small number of variants that could be assayed. Today, technology allows for the genotyping of individuals to extract sets of genomic variation and is no longer an obstacle. Usually, contemporary genotyping assays include hundreds of thousands of common genetic variants. However, common DNA variation adds only a small effect to the phenotype, and many variants are contributing. Thus, there is a need to identify lots of variants before testing them, and large sample sizes are needed to do that. However, even if we would have sequences of genomes from every person on Earth and perform a genome-wide association study (GWAS), traits could not be predicted with 100% accuracy. Statistically, there is no linear endpoint. With such a complete database, science could only tell how much of the variability in the world was due to genetics [4]. To get the complete picture of a particular trait, a consideration of the environment is necessary. Knowledge of the DNA variants that an individual carries can only predict the genetic value of the individual for a trait. Thus, the accuracy with which the phenotype is predicted from DNA variants is limited because the impact of environmental factors is ignored. However, this limitation may be overcome by combining genetic predictors with predictors of environmental influences [5].

Besides the intricacy of complex trait genetics itself and statistical challenges, the critical factor limiting the application of behavior genetic findings in practice is ethical issues. The memory of the improper application of genetic knowledge, known as eugenics, still casts a shadow on current attempts to apply behavior genetics. The discrimination regarding genetic information in various socially important aspects of life, such as employment or health insurance, is also a considerable concern. There are also common worries such as privacy and safety of personal information. Nevertheless, genetics paves the way in medicine, forensics, recreation (related to direct to consumer (DTC) tests). All we need is to work hard to integrate different disciplines, and in our case—improve computational genetic methods, advance human performance in CS based on the comprehensive knowledge we extract from different sources.

### 2.1. Determination of Behavior

**Figure 1:**
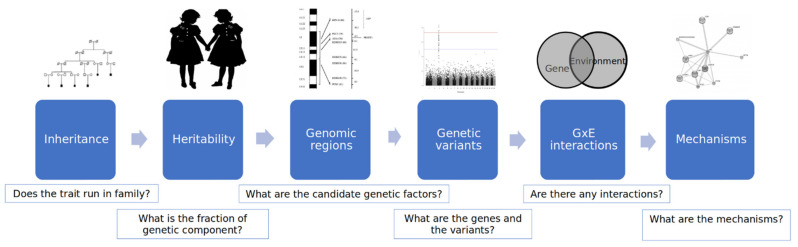
Genetic methods for the analysis of complex trait—behavior.

Probably most of us usually do not even think about how the response to scalding with boiling water is generated. The instant retreat from the boiling water is the result of genetically determined behavioral patterns. Every trait on its basis is a genetic working, and the level of trait expression depends on the environment, which shapes the genetic program. As Dr. Francis Collins (Director of the National Institutes for Health (NIH), leader of the Human Genome Project) once said: “The gene proposes, the environment disposes. Genes load the gun, but the environment pulls the trigger”. Only a few human traits are purely monogenic (i.e., determined only by one gene), most of them are multifactorial, thus as implied in the name—complex. Behavior is one of these. There are several genetic methods used to evaluate the level of genetic components that shape human behavior (Figure 1, adapted from Smoller, 2016 [6]).

Human and model organisms’ (e.g., monkeys, dogs, rodents, and other) studies are the two main types of methodologies to investigate behavioral traits. To determine whether the trait is inherited, it must run in families, and when the significant familial recurrence ratio is achieved; it can be concluded that the disorder is familial. To evaluate *h*^2^, the narrow-sense heritability [7], i.e., what fraction of a trait variation depends on genetic factors, human studies mainly involve twin (identical or monozygotic and non-identical or dizygotic) pairs or sibling analysis strategies. The point of the strategy here is that we can relatively distinguish the environmental factors from genetic factors as twins or siblings typically experience similar environments while growing up: family social status, exposure to toxins, diet, climate, etc., all tend to be similar [8,9]. Furthermore, siblings are concordant for ancestry and display negligible differences in population structure [10]. Heritability ranges from zero when there is no contribution of genetic variants to the phenotypic variation to 100% when phenotypic variation entirely depends on genetic variation (i.e., monogenic).

Once the heritability of a trait is determined, molecular genetic studies can be undertaken to map and identify the genetic factors at the level of DNA variation. For complex traits to identify genetic regions (loci), association studies are a first-choice method and more powerful than linkage studies. GWAS became a dominant strategy for many traits, disorders, and conditions. Association studies typically utilize a case-control design to determine whether specific genetic variants (alleles) are more common among the group expressing the trait (cases) than among the individuals without a trait (controls) [6]. In general, two strategies for association analysis of SNVs are widely used: candidate gene studies and genome-wide association studies (GWAS). The first relies on genes that have been implicated in a phenotype-based on prior evidence [11]. In contrast, GWAS enables a so-called ‘unbiased’ search for risk loci by examining variants across the genome instead of limiting the search to hypothesized candidates. GWAS of common SNPs became possible with the development of DNA microarrays that interrogate millions of positions across the genome. More recently, advances in DNA sequencing technologies have enabled exome-wide (and even genome-wide) analysis of rare variants. Very large sample sizes (on the order of 25,000 or more cases) are needed to adequately power genome-wide analyses of either common or rare variants [6].

After we determine underlying genetic factors, the next step would be to analyze the possible gene x gene interactions (epistasis, modifier genes) and also gene x environment (GxE) interactions. These studies examine whether the effect of a genetic variant is modified by environmental exposure.

Establishing that a genome variant, gene, or gene set is associated with a disorder, or a trait of interest is only the first step in answering the question of how specific genes contribute to the disorder or the particular trait (e.g., behavioral aspect related to a skill/characteristic which is desired/undesired for CS specialist). A wide range of molecular, cellular, and clinical research studies may be needed to characterize the pathogenic or alternative mechanisms involved. These include studies of gene expression, animal and cellular models in which genes may be experimentally altered to study functional effects, and clinical neuroscience studies (e.g., neuroimaging and neurophysiology) examining the effect of genetic variation on brain structure and function [6].

As a consequence of the heterogeneity found in nature, most of the multifactorial traits, such as behavioral traits are difficult to identify in humans. Thousands of variants can have a small effect size on the trait, and this can differ dramatically in different subgroups of individuals. Studies of distinct populations and families with extensive pedigrees added great value to gene mapping and the genetic architecture of traits. Dogs can provide an illustration of this. For example, it has been shown that the dog is a valuable resource to study the genetic architecture of behavior [12]. The strengths of dog models of complex genetics have been exploited mainly in the area of cancer [13], but recently also in behavior, e.g., fear and aggression [14], obsessive-compulsive disorders [15], diverse behavioral traits such as nerve stability, wariness, adaptability, sharpness, activity and other [16]. All of these attributes are also of interest when investigating and assessing the characteristics of CS specialists.

Once the genetic factors of a trait are mapped, then we can proceed with the models for trait prognosis, or if it is a disease/condition, estimate the risk. There are several established methods on how to predict traits, and this field is quickly evolving [5]. Currently, the prediction is usually based on sequence data (imputed or assayed) or SNP panels and/or individual variants thought to be associated with the trait. The foremost method of prediction of additive genetic values is the best linear unbiased prediction (BLUP). Besides this, there are several Bayesian methodical data on livestock [17,18,19,20] that give accuracy as high as or higher than BLUP. What is more, some heuristic methods are also commonly used. In human genetics, a standard method is called the ‘polygenic risk score’ (PRS). It has the advantage that it can be calculated from summary data (i.e., estimated SNP effects) without access to individual-level data. Another general-purpose prediction method that can be applied to SNP data is partial least squares (PLS). PLS is a linear predictor and neglects linear components of the genotypes that are of lesser importance to the prediction of the phenotype. PLS results in a similar prediction to BLUP. Machine learning methods have also been applied to prediction but do not seem to have an advantage over the linear model methods described above. These methods could include non-additive interactions between alleles and loci in the prediction of genetic value. Attempts have also been made to include these non-additive effects in conventional models, but they do not generally increase prediction accuracy [21]. Trait prognosis or risk prognosis models incorporating genetic components could be applied in various fields and applications. We believe that there are some important qualities of CS specialists that might depend on behavior that could be altered/ intervened after we have an accurate prognosis. This accuracy could be achieved by including as many as possible components to the prognosis model, and genetic factors are one of them.

### 2.2. Ethics

Modern behavior genetics studies face ethical concerns relating to the medicalization of behavior traits, mistreatment, and abuse of information for insurance or employment, social aspects of information misuse such as public discrimination, impact on law and judgment, and the risk of modern eugenics. Eugenics was rather a misunderstanding of inheritance, thinking that a single gene can account for a complex behavioral trait. Eugenicists sought to improve the human population and its gene pool by encouraging “fit” individuals to procreate (positive eugenics) and discouraging or preventing the reproduction of the “unfit” (negative eugenics).

One of the ethical issues for behavior genetics is medicalization. Sometimes medicalization of human traits is not always necessary as traits previously thought to be normal can later be presented as a deviation from the normal human population [22,23,24,25,26]. The medicalization of behavior traits can lead to another ethical concern—discrimination. The discrimination of people with particular genome variations related to behavior genetics can be found in different layers [27]. First, it can be discrimination in education when children are divided according to their intelligence and IQ. Studies have shown that genomic variation accounts for 50–80% of individual differences in reading [28,29]. Also, it was shown that arithmetic skills are at least partially genetically determined [30]. Recent studies of educational behavior and application in practice could have a positive goal—to help individuals with particular behavior to get better education/occupation. Even the initiative for genome screening related to behavior traits for achieving better education was proposed. Researchers suggested that special education from an early age could produce better learning results. However, there is a thin line not to be crossed here regarding discrimination and abuse. Second, it can be discrimination in work when an employer may not accept or fire an employee that he considers to have undesirable traits. It also can be discrimination by the insurance companies if insurers would use personality traits that are known from genome data, such as novelty-seeking to estimate risk and so increase the cost of insurance [31]. It can be discrimination in law when someone accused can be judged by one’s behavioral genome data and not directly by the crime evidence.

It is of great concern that knowledge of behavior-related variation in the genome could be misused in social and political aspects such as voting. A study by Hatemi et al. (2009) showed that there is genome variation between liberal and conservative voters in the US and that it is related to the cognitive processing of fear [32].

There has recently been concern over the use of genome profiling that is performed by DTC genetic testing companies (e.g., 23 and Me) as the results they provide are conflicting. “My Gene Profile” company declared that it was able to provide a very wide behavior profile—intelligence, emotion, artistic ability, addiction traits, and also physical performance. This company was shut down [33], and this case shows the need for tighter regulations.

Science in recent years revealed a high amount of data related to behavior genetics that can benefit the military, and this knowledge has already been in use. A good example of behavior genetics application for military purposes is the Defense Advanced Research Projects Agency (DARPA) research project of the drug called modafinil, which boosted cognitive and physical performance in soldiers [33,34].

Taking into account ethical issues, the major risks for CS field specialists might be personal genetic data leak or another mistreatment (e.g., matter for social engineering) and information abuse for a discriminative purpose (e.g., the reason for not hiring). These hazards should be carefully articulated and tackled before introducing genetic data into the practice.

### 2.3. Human behind the Scene in Cybersecurity

Human factors play an essential role in CS, and in most well-known cases, a human is either the possible source, vehicle, or destination [35] of cyber incidents. Deconstructing attribution [36] of the cyber incidents is a multifaceted cyber-physical process often split into technological, social, legal, and political dimensions. Often, the process ends in terms of indicators of compromise in threat intelligence platforms [37] or much more extensive socio-political analysis and condemnation. Hutchins et al. (2011) discuss Cyber Kill Chain by structuring cyber-attacks [38]. Such a clear view enables a structured view on the assessment of the risk and human participation in all stages of the attack. The structured view of the Cyber Kill Chain [39] enables stochastic probability models to be computationally derived and further extended.

Typically, CS professionals are viewed as smart, technically skilled individuals [40], and it is not easy to attract new talents to this professional field. There is a need to build a picture of CS work roles by de-emphasizing technical competencies and focusing on other desired skills, such as communication, decision-making, and support.

Most CS study programs follow the ACM/IEEE Cybersecurity Curricula [41] that includes several non-technical knowledge areas (human security, organizational security, and societal), as a cyber-specialist should possess knowledge in ethics, policy, law, and human vulnerability.

The NIST NICE Cybersecurity Workforce Framework [42] is one of the best worldwide known frameworks to define the working area of CS specialists. The framework covers a set of roles and maps them to knowledge, skills, and abilities. The updated NICE framework [43] introduces a concept of competence together with tasks for the role. The CS field is dynamic, and a specific workplace might require doing specific tasks. Parrish et al. (2018) emphasize that technical skills and human disposition (personal qualities, e.g., socio-emotional skills, attitude) make a much larger impact on the success of the CS professional than just knowledge itself [44]. Therefore, the intersection of knowledge, skills, and disposition describes the intended competency.

Esparza et al. (2020) consider the habituation factor in knowledge, attitude, and behavior model and support individuals’ likelihood of action by background, beliefs, and prior experiences to assess cyber hygiene-related skills [45]. Alohali et al. (2018) investigated correlations among several personality factors and security behavior [46]. For example, based on the findings, people with high neuroticism were more unstable, and their security behavior could be more radical than others. Therefore, user-oriented factors could be used to predict risk-taking behavior.

Prevention against the attacks is the best strategy in CS. Furthermore, to understand criminal behavior, it is not enough to focus on technology [47]. Moreover, the human factor should be seen as a part of the solution, not as a problem [48].

## 3. Psychological Factors

Recent research has shown that genetic heritability of personality is calculated to be 0.40, but this varies depending on which personality inventory is used and which personality factors are measured [49]. For example, Extraversion, as measured with the Eysenck scale, has a 0.42 heritability index, while the Five-factor model has 0.36.

Other psychological factors, such as cognitive styles, may also have genetic underpinnings that are relevant for CS specialist profiles. Cognitive styles, biases, and appraisals have been shown to have correlations with genetic underpinnings, while personality factors show less heritability as we age. For example, the heredity of intellect increases significantly as a person ages, whereas the heritability of personality declines slightly, and although increasing cognitive stability with age is largely mediated by genetic variables, rising personality stability with age shows mediation by environmental factors [50].

This discrepancy between personality and cognitive aspects could be due to developmental processes in childhood and adolescence. For example, personality has strong correlations to infantile temperament [51], while cognitive aspects may be more dependent on the biological development of brain areas and its connections and hormonal systems that mature as one ages [52].

A recent review has shown that there are discrepancies in genetic explanations for personality factors and that new techniques, i.e., next-generation sequencing, can help better understand the genetic contribution to personality factors [53].

Recent research on personality and cognitive factors in CS specialists is in its early stages, but findings on both personality and cognitive profiles are becoming more available [54,55].

### 3.1. Stress

We present an example to identify the potential of human factor identification based on the genetic data. We chose the stress factor (SF) as a representative human factor understandable globally and frequently used in an everyday setting.

#### 3.1.1. Stress Factor Defined by Genome

Stress is defined as a state of threatened homeostasis that evokes a multiplicity of somatic and adaptive reactions [56,57]. As with other human behavioral traits, stress has complex manifestations due to genomic and environmental factors.

The role of stressful environments and the physiology of stress response systems have been most closely linked to depression, anxiety, and traumatic stress disorders^6^. How do we identify those individuals who are more susceptible to stress? This knowledge is important for an individual when choosing a profession or place of work, as well as for the institution hiring a specialist. For the more susceptible ones, stressful working conditions such as flight control or CS might be too difficult to cope with. Studies are showing that individuals might differ substantially according to how they respond to similar experiences. It has been demonstrated that environmental sensitivity depends equally on genetic factors as well as on environmental factors and that there are overlaps between personality traits of neuroticism and extraversion [2]. Also, as an example, it has been shown that the evaluation of the glucocorticoid receptor gene variant could help to identify children differentially susceptible to stress and intervention to overcome adverse negative environmental effects. Thus, information on the genetic background could be valuable if we would like to influence certain behavior or a good indicator of whether we should invest money and time in that person [58].

**Figure 2:**
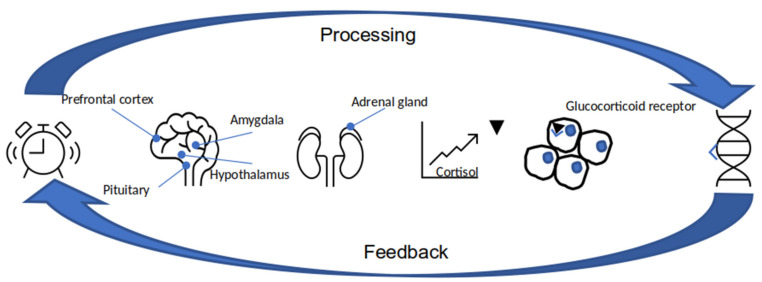
Pathway showing stress and DNA relation.

It is already well known that cortisol, which is produced in the adrenal gland, is the main stress hormone. After a person experiences a stressful event, the level of glucocorticoid rises in the blood. The prefrontal cortex reacts to stress by making things look less scary, the amygdala identifies danger from the environment. Then, the hypothalamus activates the pituitary gland, which synthesizes hormones and induces the adrenal glands to produce cortisol. Released into the bloodstream, cortisol reaches cells, binds to the intracellular glucocorticoid receptors, and the receptors change, then translocate to the nucleus where interact with the DNA [59]. Figure 2 shows the physical effect of stress.

Cortisol is a stress response system that increases heart rate and breathing, provides energy to the muscles, allows clear thinking, and may boost memory. Cortisol shifts the balance of neurotransmitters. It lowers dopamine that reduces the activity of the pleasure pathway, lowers norepinephrine which reduces motivation, and lowers serotonin which reduces the mood.

Twins studies revealed 69–72% genetic impact on cortisol level [60,61]. Genes related to cortisol, dopamine, serotonin, and norepinephrine directly affect the stress response. In addition, genes that control neurotransmitters serve as neurotransmitter modulators that participate in neuronal plasticity and stabilize synapses. Genome-wide association studies provided many genes associated with stress like *GR*, *NR3C1*, *GABRA6*, *OPRM1*, *ACE*, *FKBP5*, *GCCR*, *CNR1*, *DRD2*, *ANKK1*, *NPY*, *DBH* [56,62]. However, there are known particular genes and SNPs in them that have a significant impact on stress resistance and response during it. One of them is the *COMT* gene, whose main function is to regulate the production of dopamine and affect how decisions are being made under pressure, control stress, and resiliency [63]. Other authors refer to this gene as “warrior/worrier”, because this gene variant shows how a person responds to stress [64,65]. For a “warrior” person stress has less impact, and the person may even benefit from it. A “worrier” person has lower stress resiliency. The high impact of the *COMT* gene has been proved not only in human but also animal model studies such as those on rats or primates [65,66]. For example, Pflüger et al. (2016) proved the high *COMT* gene impact to stress by studying Japanese macaques, which have a high level of aggressive interaction and have a different response to stressful events [66]. Another gene related to stress is *BDNF*, which serves as a neurotransmitter modulator, participates in neuronal plasticity, and stabilizes synapses [63]. This gene has been shown to relate to stress in human and animal studies [67,68,69]. People with specific variants in *BDNF* have better stress resilience. The final gene worth mentioning related to stress is *SLC6A4*, which is the serotonin transporter gene. Serotonin is known as the mood neurotransmitter and has been well-studied for depression, suicide, and stress [70,71]. It is known that these genes are related to stress, and those specific variants of these genes have a high impact—rs4680 variant on *COMT* gene, rs6265 variant on *BDNF* gene, and rs25531 on *SLC6A4* gene [65,70,72]. For example, Qi et al. (2020) studied post-traumatic stress disorder by analyzing genome data of the parents in China that lost their only child [72]. Not all parents who lost their only child suffered from a posttraumatic stress disorder, and rs4680 (*COMT*), rs6265 (*BDNF*) have an impact on this [72].

The specific genome variants allow using this knowledge for practical purposes without extensive, expensive, and lengthy analysis studies. Furthermore, these variants are already being used by private companies for their customers to learn more about their genome variation. Knowing stress-related genes may be used for sports, education, or finding a more suitable occupation. Vogel and Schwabe (2016) studied the learning process under the stress condition and found that there was a different effect on different people, but the administration of 20 mg of cortisol increased memory and learning process rate [73]. The “warrior” personality type is better at picking highly stressful jobs, and learning under stressful conditions even has benefits like memory boost.

Twin studies on the heritability of different neurotransmitters have uncovered differentiating aspects of genetic involvement. For example, genetic influences on cortisol have been shown to have high heritability at younger ages for cortisol production, and this decreases during development [74]. They showed that the broad-sense heritability index for cortisol production went from 42% at the age of 9 decreased to 0% at the age of 17 indicating that environmental factors could better explain cortisol production. The same study also showed that cortisol metabolism during the same age span showed that A-ring reductases, which has both pubertal enzyme regulation and cortisol metabolism functions, showed stable or increased genetic heritability, from 0–23% at the age of 9, from 23% to 51% at the age of 12, and from 51% to 66% at the age of 17 [75]. The differences in production and metabolism during development and puberty have long lasting influences on the development of the HPA -axis, indicating that genetic influence initially influences cortisol production but is then more influenced by environmental aspects, while cortisol metabolism heritability increases with age [75]. Studies have also shown that suppressed expression of A-ring reductases in the HPA-axis contributes to increasing illnesses [76]. The HPA-axis is influenced by the anterior cingulate cortex (ACC). The ACC is involved when effort and regulatory processes are necessary for attention, effortful control, and problem-solving [77] and has been shown to have genetic heritability [78]. The Anterior Attention System, which involves the ACC and adjacent mid-prefrontal cortex, has been shown to modulate stress systems, i.e., HPA-axis [79]. Decreased grey matter volume in the ACC has also been shown to have an association with depression and increased critical illness through decreased cortisol metabolization [80,81], while decreased ACC connectivity has been shown to adversely modulate stress responses and psychophysiological wellbeing [82].

#### 3.1.2. Stress in Cybersecurity Professional Career

The document of ACM/IEEE Cybersecurity Curricula [41] does not have the word *stress* mentioned at all. 

The word *stress* is not found in the NIST NICE framework [42] either. However, the term *time* (or timely) is used to define stressful conditions that require urgent decisions and sharing to authorities (or administration) at the right time. Table 1 provides several task examples with their descriptions and associated work roles. For example, a person in a multi-disciplinary work role.

Language analysts manipulate time-sensitive information to inform authorities (T0854). Threat/Warning analysts and all-source analysts should provide timely notice about hostile activities, as delay would mean higher cost (T0800). Cyber defense analysts should detect and alert on time about anomalous activities (T0258).

In ACM/IEEE Cybersecurity Curricula, concept *time* is related to the analysis of timelines, checking states of data/objects, real-time monitoring (or controls), network analysis, project management, time management in social media, and agile decision making. Nevertheless, time pressure—the stress factor, is missing. In the context of higher education, the critical aspect is learning what and how to look for tools and develop abilities to combine data sources, while professionals have to deliver tasks on time with an impact of the decision in mind.

The ACM/IEEE Cybersecurity Curricula and the NIST NICE Framework do not reflect the stress factor that is very common in CS work roles. Oltsik (2019) reports that 13% and 33% of CS specialists strongly agree or disagree, respectively, that they feel an unhealthy level of stress as part of the job [83]. Other surveys report that 62% of professionals feel stressed or very stressed by their jobs [84]. Among all stress aspects, there are factors like the overwhelming workload, keeping up regulatory compliance audits, the fear of getting something wrong, and constant emergencies and disruptions [83]. Therefore, determination of the personal profile towards stress management could overall impact personal resilience against cyber incidents.

The standard CS workforce preparation provides the basic skills required to enter positions as junior specialists [84], but there is a gap between skill development in higher education and skill usage in work positions. Personal traits are obtained from the social environment or defined in the genome that shapes the possible future professional. An employer focuses on achieving the best performance of the organization by selecting the most advantageous working staff in CS. The employer applies an indirect personal trait analysis [85,86] for better employee integration into the organization. In most cases, personal traits that are beneficial to support the best performance of CS specialists are not considered and become apparent after a period of time.

As an example, we can deconstruct the following real CS situation. Objectives “Identify indicators of compromise (IoCs) using threat detection tools” and “Negotiate IoCs with collaborators” could be associated with a competence “Management and sharing of threat information” because the competence requires the collection of IoCs from the tools, a collaboration between team members during IoC retrieval process, and defining IoCs in the reports. The competence can be related to the task shortly defined as “Timely information management”. The person should be stress-resistant as the task requires:

- communication with collaborators that are busy with an incident response (their primary focus is not reporting);
- systemic thinking (IoCs must be correct and context-related);
- self-control (the report must be submitted in time).

Therefore, it would be beneficial to identify the stress-related risk level of a particular person. The genomic analysis could be a complementary part of the assessment as it encompasses the information on the natural traits of the person. The personal risk makes an impact on the performance indicators of the person and the team itself.

## 4. Concluding Remarks

This paper describes how human factors could be identified from the genetic data and used for personalized risk assessment taking stress as a case. The current competence frameworks do not include personalized approaches. Integrating human behavior factors identified from genomic data into risk assessment strategies and professional training outside the standard IT-oriented training schema is a thrilling challenge but with great additional value. We chose the sufficiently genetically reasoned stress factor to emphasize the impact of genetic information on performance in the CS field. Stress creates situations when humans behave not as trained but naturally, based on genetic traits. Therefore, various human features that can be defined by genetic information could be important risk factors, e.g., addiction, introversion, aggressive behavior, depression, post-traumatic stress disorder. Integration of genomic data into CS provides new opportunities to support an individualized approach. For a particular person learning his/her genome variants related to human behavior can help to expose and accept his/her strengths and weaknesses to use and/or overcome them later. The provided example of stress-related genome variants could guide the person on how to affect one’s reaction to the situations and circumstances caused by stress, demonstrate the level of learning process under stress conditions, and show resistance to stress.

Next to new approaches to CS, a number of challenges arise with their application. Firstly, human genome data are considered highly sensitive data. Data administration should follow procedures to ensure restricted access and high-level security, including both raw, derived, and aggregated data associated with a person or any incident response team. Secondly, interpretation of data and personalized risk assessment based on genomic data should be performed carefully and ethically with many additional factors in mind. The risk assessment cannot be based solely on genetic data to assign a person for a work role. Any employee should be protected by legal documents against discrimination based on findings from genetic data. Finally, to integrate genomic data into the CS exercises requires that challenges in human and technological resources are overcome. 

Multicomplexity/multifactoriality of the discussed research field—the behavioral genetics of the aspects of the cybersecurity specialists’ activities is the main challenge in identifying the wide combination of most possible factors. The ever-increasing number of GWAS hits harness the power of molecular genetics to identify specific genes responsible for genetic influence on reacting to environmental triggers, such as stress. Future research findings will be utilized for the analytics of the prediction scores. As well the delineation of specific combinations of genomic factors and environmental factors (or construct of gene-gene and gene-environment landscape plot/map) will suggest the best calculation methods and attempt for ranging those prediction scores. The work can be extended in several directions. Pilot studies should be executed to evaluate exercise implementation and collect quantitative data for further research. Behavioral Neural Networks should also be addressed to identify the risks detection process and the needs for behavioral references. Risk assessment strategies with more precise parameter settings should be built with additional risks in mind. Evaluation of ethically concerning aspects and determination of measures have to be taken in order to tackle ethics-related risk management. 

## Figures and Tables

**Table 1:** Examples of tasks with some emphasis on stressful conditions due to time limits.

Task	Description	Role
T0854	Tip critical or time-sensitive information to appropriate customers	Multi-Disciplined Language Analyst
T0800	Provide timely notice of imminent or hostile intentions or activities which may impact organization objectives, resources, or capabilities	Threat/Warning Analyst, All-Source Analyst
T0258	Provide timely detection, identification, and alerting of possible attacks/intrusions, anomalous activities, and misuse activities and distinguish these incidents and events from benign activities	Cyber Defense Analyst

## Data Availability

Not applicable.

## References

[1] Human Genome Project Information Archive 1990–2003. http://web.ornl.gov/sci/techresources/Human_Genome.

[2] Assary E., Zavos H.M.S., Krapohl E., Keers R., Pluess M. (2020). Genetic architecture of Environmental Sensitivity reflects multiple heritable components: A twin study with adolescents. Mol. Psychiatry.

[3] Insel T.R. (2013). Brain somatic mutations: The dark matter of psychiatric genetics?. Mol. Psychiatry.

[4] Division of Behavioral and Social Sciences and Education (2017). Social and Behavioral Sciences for National Security.

[5] Balding D.J., Moltke I., Marioni J. (2019). Handbook of Statistical Genomics.

[6] Smoller J.W. (2016). The Genetics of Stress-Related Disorders: PTSD, Depression, and Anxiety Disorders. Neuropsychopharmacology.

[7] Visscher P.M., Hill W.G., Wray N.R. (2008). Heritability in the genomics era—Concepts and misconceptions. Nat. Rev. Genet..

[8] Felson J. (2014). What can we learn from twin studies? A comprehensive evaluation of the equal environments assumption. Soc. Sci. Res..

[9] Jelenkovic A., Sund R., Hur Y.-M., Yokoyama Y., Hjelmborg J., Möller S., Honda C., Magnusson P., Pedersen N., Ooki S. (2016). Genetic and environmental influences on height from infancy to early adulthood: An individual-based pooled analysis of 45 twin cohorts. Sci. Rep..

[10] Lello L., Raben T.G., Hsu S.D.H. (2020). Sibling validation of polygenic risk scores and complex trait prediction. Sci. Rep..

[11] Sullivan P.F. (2007). Spurious Genetic Associations. Biol. Psychiatry.

[12] Friedrich J., Strandberg E., Arvelius P., Sánchez-Molano E., Pong-Wong R., Hickey J.M., Haskell M.J., Wiener P. (2019). Genetic dissection of complex behaviour traits in German Shepherd dogs. Heredity.

[13] Alvarez C.E. (2014). Naturally Occurring Cancers in Dogs: Insights for Translational Genetics and Medicine. ILAR J..

[14] Zapata I., Serpell J.A., Alvarez C.E. (2016). Genetic mapping of canine fear and aggression. BMC Genom..

[15] Tang R., Noh H.J., Wang D., Sigurdsson S., Swofford R., Perloski M., Duxbury M., Patterson E.E., Albright J., Castelhano M. (2014). Candidate genes and functional noncoding variants identified in a canine model of obsessive-compulsive disorder. Genome Biol..

[16] Ha J.H., Alam M., Lee D.H., Kim J.-J. (2015). Whole Genome Association Study to Detect Single Nucleotide Polymorphisms for Behavior in Sapsaree Dog (Canis familiaris). Asian-Aust. J. Anim. Sci..

[17] Erbe M., Hayes B., Matukumalli L., Goswami S., Bowman P., Reich C., Mason B., Goddard M. (2012). Improving accuracy of genomic predictions within and between dairy cattle breeds with imputed high-density single nucleotide polymorphism panels. J. Dairy Sci..

[18] Kemper E.K., Reich C.M., Bowman P.J., Jagt C.J.V., Chamberlain A.J., Mason A.B., Hayes B.J., Goddard M. (2015). Improved precision of QTL mapping using a nonlinear Bayesian method in a multi-breed population leads to greater accuracy of across-breed genomic predictions. Genet. Sel. Evol..

[19] MacLeod I.M., Bowman P.J., Jagt C.J.V., Haile-Mariam M., Kemper K.E., Chamberlain A.J., Schrooten C., Hayes B.J., Goddard M.E. (2016). Exploiting biological priors and sequence variants enhances QTL discovery and genomic prediction of complex traits. BMC Genom..

[20] Moser G., Lee S.H., Hayes B., Goddard M., Wray N.R., Visscher P. (2015). Simultaneous Discovery, Estimation and Prediction Analysis of Complex Traits Using a Bayesian Mixture Model. PLoS Genet..

[21] Aliloo H., Pryce J.E., González-Recio O., Cocks B.G., Hayes B.J. (2016). Accounting for dominance to improve genomic evaluations of dairy cows for fertility and milk production traits. Genet. Sel. Evol..

[22] Clark J. (2014). Medicalization of global health 1: Has the global health agenda become too medicalized?. Glob. Health Action.

[23] Hart G. (2002). Sexual behaviour and its medicalisation: In sickness and in health. BMJ.

[24] Nuffield Council on Bioethics (2003). Genetics and Human Behaviour: The Ethical Context–Summary and Recommendations. J. Int. Bioeth. Int. J. Bioeth..

[25] Psychiatry T.L. (2016). Medicalisation and its discontents. Lancet Psychiatry.

[26] Wyatt W.J. (2009). Behavior Analysis in the Era of Medicalization: The State of the Science and Recommendations for Practitioners. Behav. Anal. Pr..

[27] Rothstein A.M., Anderlik M.R. (2001). What is genetic discrimination, and when and how can it be prevented?. Genet. Med..

[28] Byrne B., Olson R.K., Samuelsson S., Wadsworth S., Corley R., DeFries J.C., Willcutt E. (2006). Genetic and environmental influences on early literacy. J. Res. Read..

[29] Petrill S.A. (2016). Behavioural Genetic Studies of Reading and Mathematics Skills. Behav. Genet. Educ..

[30] Libertus M.E., Feigenson L., Halberda J. (2011). Preschool acuity of the approximate number system correlates with school math ability. Dev. Sci..

[31] Klitzman R. (2010). Views of Discrimination among Individuals Confronting Genetic Disease. J. Genet. Couns..

[32] Hatemi P., Alford J.R., Hibbing J.R., Martin N., Eaves L.J. (2009). Is There a “Party” in Your Genes?. Politi-Res. Q..

[33] Berryessa C.M., Cho M.K. (2013). Ethical, Legal, Social, and Policy Implications of Behavioral Genetics. Annu. Rev. Genom. Hum. Genet..

[34] Puscas I.M. (2020). Military Enhancement: Technologies, Ethics and Operational Issues. Ethics of Medical Innovation, Experimentation, and Enhancement in Military and Humanitarian Contexts.

[35] Lemay A., Calvet J., Menet F., Fernandez J.M. (2018). Survey of publicly available reports on advanced persistent threat actors. Comput. Secur..

[36] Saalbach K.-P. (2019). Attribution of Cyber Attacks. Inf. Technol. Peace Secur..

[37] MISP Project (2019). MISP—Open Source Threat Intelligence Platform & Open Standards For Threat Information Sharing. https://www.misp-project.org.

[38] Hutchins E.M., Cloppert M.J., Amin R.M. (2011). Intelligence-driven computer network defense informed by analysis of adversary campaigns and intrusion kill chains. Lead Issues Inf. Warf Secur. Res..

[39] Hoffmann R., Saeed K., Dvorský J. (2020). Stochastic Model of the Simple Cyber Kill Chain: Cyber Attack Process as a Regenerative Process. Computer Information Systems and Industrial Management.

[40] ISC22 (2020). How Views on Cybersecurity Professionals Are Changing and What Hiring Organizations Need to Know. The 2020 (ISC)2 Cybersecurity Perception Study.

[41] ACM (2017). Joint Task Force on Cybersecurity Education. Cybersecurity Curricula 2017: Curriculum Guidelines for Post-Secondary Degree Programs in Cybersecurity.

[42] Newhouse W., Keith S., Scribner B., Witte G. (2017). National Initiative for Cybersecurity Education (NICE) Cybersecurity Workforce Framework. NIST Spec. Publ..

[43] Petersen R., Santos D., Smith M.C., Wetzel K.A., Witte G. (2020). Workforce Framework for Cybersecurity (NICE Framework.

[44] Parrish A., Impagliazzo J., Raj R.K., Santos H., Asghar M.R., Jøsang A., Pereira T., Stavrou E. Global perspectives on cybersecurity education for 2030: A case for a meta-discipline. Proceedings of the Companion of the 23rd Annual ACM Conference on Innovation and Technology in Computer Science Education.

[45] Esparza J., Caporusso N., Walters A., Corradini I., Nardelli E., Ahram T. (2020). Addressing Human Factors in the Design of Cyber Hygiene Self-assessment Tools. Advances in Human Factors in Cybersecurity.

[46] Alohali M., Clarke N., Li F., Furnell S. (2018). Identifying and predicting the factors affecting end-users’ risk-taking behavior. Inf. Comput. Secur..

[47] Corradini I. (2020). Redefining the Approach to Cybersecurity.

[48] Zimmermann V., Renaud K. (2019). Moving from a ‘human-as-problem” to a ‘human-as-solution” cybersecurity mindset. Int. J. Human-Comput. Stud..

[49] Vukasović T., Bratko D. (2015). Heritability of personality: A meta-analysis of behavior genetic studies. Psychol. Bull..

[50] Briley D.A., Tucker-Drob E.M. (2017). Comparing the Developmental Genetics of Cognition and Personality over the Life Span. J. Pers..

[51] Rothbart M.K., Ahadi S.A., Evans D.E. (2000). Temperament and personality: Origins and outcomes. J. Pers. Soc. Psychol..

[52] Savitz J., Solms M., Ramesar R. (2006). The molecular genetics of cognition: Dopamine, COMT and BDNF. Genes Brain Behav..

[53] Zmorzyński S., Styk W., Klinkosz W., Iskra J., Filip A.A. (2021). Personality traits and polymorphisms of genes coding neurotransmitter receptors or transporters: Review of single gene and genome-wide association studies. Ann. Gen. Psychiatry.

[54] Freed S.E. (2014). Examination of Personality Characteristics Among Cybersecurity and Information Technology Professionals.

[55] Lugo R.G., Sütterlin S. (2018). Cyber Officer Profiles and Performance Factors. Lecture Notes in Computer Science.

[56] Ising M., Holsboer F. (2006). Genetics of stress response and stress-related disorders. Dialog-Clin. Neurosci..

[57] Pacák K., Palkovits M. (2001). Stressor Specificity of Central Neuroendocrine Responses: Implications for Stress-Related Disorders. Endocr. Rev..

[58] Albert D., Belsky D.W., Crowley D.M., Latendresse S.J., Aliev F., Riley B.P., Sun C., Dick D.M., Dodge K.A. (2015). Conduct Problems Prevention Research Group Can Genetics Predict Response to Complex Behavioral Interventions? Evidence from a Genetic Analysis of the Fast Track Randomized Control Trial. J. Policy Anal. Manag..

[59] Allen M.J., Sharma S. (2020). Physiology, Adrenocorticotropic Hormone (ACTH).

[60] Riese H., Rijsdijk F.V., Rosmalen J., Snieder H., Ormel J. (2009). Neuroticism and Morning Cortisol Secretion: Both Heritable, But No Shared Genetic Influences. J. Pers..

[61] Rietschel L., Streit F., Zhu G., McAloney K., Frank J., Couvy-Duchesne B., Witt S.H., Binz T., McGrath J., CORtisolNETwork (CORNET) Consortium (2017). Hair Cortisol in Twins: Heritability and Genetic Overlap with Psychological Variables and Stress-System Genes. Sci. Rep..

[62] Cornelis M.C., Nugent N., Amstadter A.B., Koenen K.C. (2010). Genetics of Post-Traumatic Stress Disorder: Review and Recommendations for Genome-Wide Association Studies. Curr. Psychiatry Rep..

[63] Bathina S., Das U.N. (2015). Brain-derived neurotrophic factor and its clinical implications. Arch. Med. Sci..

[64] Goldman-Rakic P.S., Muly E.C., Williams G.V. (2000). D_1_ receptors in prefrontal cells and circuits. Brain Res. Rev..

[65] Tartar J.L., Cabrera D., Knafo S., Thomas J.D., Antonio J., Peacock C.A. (2020). The “Warrior” COMT Val/Met Genotype Occurs in Greater Frequencies in Mixed Martial Arts Fighters Relative to Controls. J Sports Sci Med.

[66] Pflüger L.S., Gutleb D.R., Hofer M., Fieder M., Wallner B., Steinborn R. (2016). Allelic variation of the COMT gene in a despotic primate society: A haplotype is related to cortisol excretion in Macaca fuscata. Horm. Behav..

[67] Cattaneo A., Cattane N., Begni V., Pariante C.M., Riva M.A. (2016). The human BDNF gene: Peripheral gene expression and protein levels as biomarkers for psychiatric disorders. Transl. Psychiatry.

[68] Lubin F.D., Roth T.L., Sweatt J.D. (2008). Epigenetic Regulation of bdnf Gene Transcription in the Consolidation of Fear Memory. J. Neurosci..

[69] Van Winkel M., Peeters F., van Winkel R., Kenis G., Collip D., Geschwind N., Jacobs N., Derom C., Thiery E., van Os J. (2014). Impact of variation in the BDNF gene on social stress sensitivity and the buffering impact of positive emotions: Replication and extension of a gene–environment interaction. Eur. Neuropsychopharmacol..

[70] Duman A.E., Canli T. (2015). Influence of life stress, 5-HTTLPR genotype, and SLC6A4 methylation on gene expression and stress response in healthy Caucasian males. Biol. Mood Anxiety Disord..

[71] Risch N., Herrell R., Lehner T., Liang K.-Y., Eaves L., Hoh J., Griem A., Kovacs M., Ott J., Merikangas K.R. (2009). Interaction Between the Serotonin Transporter Gene (5-HTTLPR), Stressful Life Events, and Risk of Depression. JAMA.

[72] Qi R., Luo Y., Zhang L., Weng Y., Surento W., Li L., Cao Z., Lu G.M. (2020). Effects of COMT rs4680 and BDNF rs6265 polymorphisms on brain degree centrality in Han Chinese adults who lost their only child. Transl. Psychiatry.

[73] Vogel S., Schwabe L. (2016). Learning and memory under stress: Implications for the classroom. NPJ Sci. Learn..

[74] Van Keulen B.J., Dolan C.V., Andrew R., Walker B.R., Pol H.E.H., Boomsma D.I., Rotteveel J., Finken M.J.J. (2019). Heritability of Cortisol Production and Metabolism Throughout Adolescence. J. Clin. Endocrinol. Metab..

[75] Van Keulen B.J., Dolan C.V., Andrew R., Walker B.R., Pol H.E.H., Boomsma D.I., Rotteveel J., Finken M.J.J. (2020). Long-Term Stability of Cortisol Production and Metabolism Throughout Adolescence: Longitudinal Twin Study. Twin Res. Hum. Genet..

[76] Boonen E., Vervenne H., Meersseman P., Andrew R., Mortier L., Declercq P.E., Vanwijngaerden Y.-M., Spriet I., Wouters P.J., Perre S.V. (2013). Reduced Cortisol Metabolism during Critical Illness. N. Engl. J. Med..

[77] Allman J.M., Hakeem A., Erwin J.M., Nimchinsky E., Hof P. (2001). The anterior cingulate cortex. The evolution of an interface between emotion and cognition. Ann. N. Y. Acad. Sci..

[78] Fan J., Wu Y., Fossella A.J., Posner I.M. (2001). Assessing the heritability of attentional networks. BMC Neurosci..

[79] Davis E.P., Bruce J., Gunnar M.R. (2001). The anterior attention network: Associations with temperament and neuroendocrine activity in 6-year-old children. Dev. Psychobiol..

[80] Liu J., Xu X., Luo Q., Luo Y., Chen Y., Lui S., Wu M., Zhu H., Kemp G., Gong Q. (2017). Brain grey matter volume alterations associated with antidepressant response in major depressive disorder. Sci. Rep..

[81] Treadway M.T., Grant M.M., Ding Z., Hollon S.D., Gore J.C., Shelton R.C. (2009). Early Adverse Events, HPA Activity and Rostral Anterior Cingulate Volume in MDD. PLoS ONE.

[82] Thomason M.E., Hamilton J.P., Gotlib I.H. (2011). Stress-induced activation of the HPA axis predicts connectivity between subgenual cingulate and salience network during rest in adolescents. J. Child. Psychol. Psychiatry.

[83] Oltsik J. (2018). The Life and Times of Cybersecurity Professionals. https://www.esg-global.com/hubfs/pdf/ESG-ISSA-Research-Report-Life-of-Cybersecurity-Professionals-Apr-2019.pdf.

[84] Exabeam (2019). Exabeam 2019: Cybersecurity Professionals Salary, Skills, and Stress Survey. https://www.exabeam.com/library/2019-cybersecurity-professionals-salary-skills-and-stress-survey.

[85] Feher A., Vernon P.A. (2021). Looking beyond the Big Five: A selective review of alternatives to the Big Five model of personality. Pers. Individ. Differ..

[86] Roberts R., Woodman T. (2017). Personality and performance: Moving beyond the Big 5. Curr. Opin. Psychol..

